# Isolation of Antimicrobial Genes from *Oryza rufipogon* Griff by Using a *Bacillus* *subtilis* Expression System with Potential Antimicrobial Activities

**DOI:** 10.3390/ijms21228722

**Published:** 2020-11-18

**Authors:** Jiale Li, Samiul Islam, Pengfei Guo, Xiaoqing Hu, Wubei Dong

**Affiliations:** Department of Plant Pathology, College of Plant Science and Technology and the Key Lab of Crop Disease Monitoring & Safety Control in Hubei Province, Huazhong Agricultural University, Wuhan 430070, China; ljl0410@webmail.hzau.edu.cn (J.L.); samiulislam@webmail.hzau.edu.cn (S.I.); GPF@webmail.hzau.edu.cn (P.G.); hxq123@webmail.hzau.edu.cn (X.H.)

**Keywords:** antimicrobial peptides, *Bacillus subtilis*, wild rice, antimicrobial potentiality, membrane permeability

## Abstract

Antimicrobial genes are distributed in all forms of life and provide a primary defensive shield due to their unique broad-spectrum resistance activities. To better isolate these genes, we used the *Bacillus subtilis* expression system as the host cells to build *Oryza rufipogon* Griff cDNA libraries and screen potential candidate genes from the library at higher flux using built-in indicator bacteria. We observed that the antimicrobial peptides OrR214 and OrR935 have strong antimicrobial activity against a variety of Gram-positive and Gram-negative bacteria, as well as several fungal pathogens. Owing to their high thermal and enzymatic stabilities, these two peptides can also be used as field biocontrol agents. Furthermore, we also found that the peptide OrR214 (MIC 7.7–10.7 μM) can strongly inhibit bacterial growth compared to polymyxin B (MIC 5–25 μM) and OrR935 (MIC 33–44 μM). The cell flow analysis, reactive oxygen burst, and electron microscopy (scanning and transmission electron microscopy) observations showed that the cell membranes were targeted by peptides OrR214 and OrR935, which revealed the mode of action of bacteriostasis. Moreover, the hemolytic activity, toxicity, and salt sensitivity experiments demonstrated that these two peptides might have the potential to be used for clinical applications. Overall, OrR214 and OrR935 antimicrobial peptides have a high-throughput bacteriostatic activity that acts as a new form of antimicrobial agent and can be used as a raw material in the field of drug development.

## 1. Introduction

The effects of resistance to antibiotics are extremely severe and may have a substantial impact on incidence and death, leading to higher economic costs for healthcare systems. Unfortunately, the development and rapid use of antibiotics have led to the gradual emergence of antibiotic-resistant strains. Therefore, an increasing number of infections are caused by microorganisms that do not respond to traditional drugs [1]. However, with the discovery of antibiotics in the twentieth century, healthcare professionals assumed that the battle with microbes was promising, but due to the emergence of a large number of antibiotic-resistant microbes, the fight intensified [2,3,4]. Hence, it is required to develop alternative antimicrobials or active compounds that offer promising therapeutic substitutes in multiple diseases related to plants, animals, and humans [5,6]. Antimicrobial genes are commonly used in agriculture because they can enhance resistance to plant diseases and also play an important role in improving crop variety [7]. Some recent studies have shown that antimicrobial peptides (AMPs) such as AtR472 derived from *Aegilops tauschii Cosson*, the cationic antimicrobial gene HCAP-18 expressed in Chinese cabbage, and peptide SM-985 from Teosinte (*Zea mays* ssp. mexicana) have significant inhibitory effects on many plant pathogenic bacteria [8,9,10]. Besides, antimicrobial peptides have also been used in many fields because of their excellent bacteriostatic effects [11,12,13,14]. In some cases, they showed anti-inflammatory and other host-defense features [15,16].

AMPs are typically produced when challenged by microorganisms and play an essential role in the innate immune system [17,18,19]. As an important part of the innate immune system, AMPs are recognized as the first defense system against the invasion of foreign pathogens by animals, plants and some other organisms [16,20,21,22]. AMPs are small oligopeptides encoded by genes (less than 100 amino acids) and often contain α helix or β sheet folding structures. They have demonstrated activities against several fungal and bacterial pathogens and even some viruses [20,21,22,23,24,25]. In addition, AMPs have the following features: broad antibacterial spectrum, fast ability to destroy germs, minimal bactericidal concentration, the capacity to be used alone or in combination, and lower side effects [15].

These outstanding features have encouraged researchers to develop techniques to isolate and purify new classes of AMPs and their derivatives from different species [26,27]. Most of the conventional methods of gene screening are focused on genetic homology, which is not an appropriate methodology for acquiring new genes [28]. Therefore, researchers are actively trying to find a new screening method that is more conducive to isolating AMPs. At present, the construction of prokaryotic and eukaryotic expression systems is gradually improving [29,30]. However, in the current state of development, the most commonly used expression system is the *Escherichia coli* (prokaryotic) expression system [31,32]. To secrete peptides into the extracellular space, the emerging *Bacillus subtilis* system can rely on its high-efficiency secretory expression. It also has a relatively perfect pathogen-free mechanism of action and is more suitable for the large-scale development of antimicrobial genes [27,33].

Rice is the main food crop in many countries around the world. There are two kinds of cultivated rice, Asian and African cultivated rice [34,35]. However, due to artificially overtargeted selection, rice has lost its fundamental resistance genes in the process of acclimation. To improve the resistance of cultivated rice, researchers are more inclined to screen resistant genes from wild-type rice [36,37]. The Zhangpu wild rice (the original ancestor of cultivated rice), which was discovered in Fujian, similar to other common wild rice, has many potential resistance genes, including resistance to disease, insects and heat [38]. At present, researchers have isolated many disease resistance genes from wild rice, and more than 20 resistance genes against rice blast have been cloned, such as *Pi*9, *Pi*21, and *Pi*54, along with genes that confer resistance to xanthophyte, such as *Xa*21, *Xa*23, and *Xa*27 [39,40,41,42].

In our lab, using a *B. subtilis* expression system, we introduced a new method for isolating resistance genes [27,43]. This approach is efficient against ‘superbug’ bacteria and different plant microorganisms for antimicrobial gene isolation. We used this technique in this research and identified resistance genes from *O. rufipogon*. In addition, the working mechanism of our isolated AMPs on pathogenic microorganisms was also preliminarily clarified, providing a theoretical basis for the subsequent analysis of the action of *O. rufipogon* resistance genes. Finally, this study also demonstrated the effectiveness of these AMPs in controlling plant pathogens.

## 2. Results

### 2.1. Screening of Antimicrobial Genes from O. Rufipogon cDNA Libraries by the B. subtilis System

**Figure 1:**
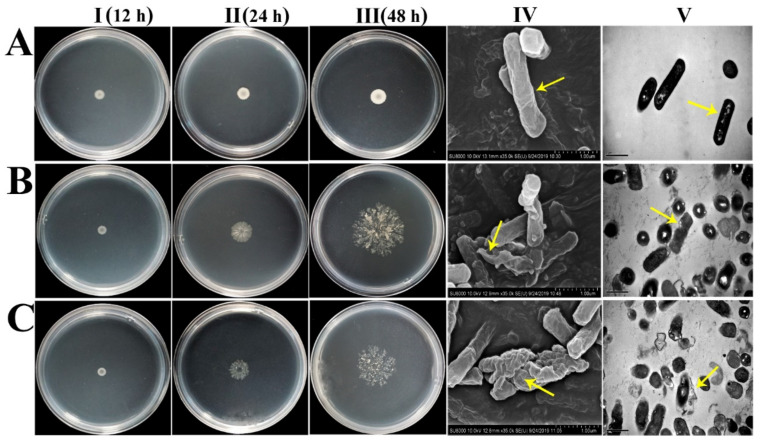
The harmful impacts of antimicrobial gene expression on host cells of *B. subtilis*. The autolytic strains (*OrR214* and *OrR935*) were placed on Luria-Bertani (LB) plates within 12 to 48 h with the *B. subtilis* SCK6-e strain and then incubated at 37 °C to observe the morphological changes. (**A**-**I**) *B. subtilis* SCK6-e strain, (**B**-**I**) *OrR935* and (**C**-**I**) *OrR214* grew normally within 12 h, and (**A**-**II**) *B. subtilis* SCK6-e strain, (**B**-**II**) *OrR935*, and (**C**-**II**) *OrR214* grew after 24 h of incubation, while (**A**-**III**) *B. subtilis* SCK6-e strain, (**B**-**III**) *OrR935*, and (**C**-**III**) *OrR214* grew after 48 h of incubation. (**B**) (*OrR935*) and (**C**) (*OrR214*) show obvious autolysis and outward diffusion. The samples were freeze-dried with glutaraldehyde and then coated with gold, and (**A**-**IV**) *B. subtilis* SCK6-e strain, (**B**-**IV**) *OrR935*, and (**C**-**IV**) *OrR214* were examined under a scanning electron microscope, and (**A**-**V**) *B. subtilis* SCK6-e strain, (**B**-**V**) *OrR935*, and (**C**-**V**) (*OrR214*) were examined under a transmission electron microscope. As a control, the *B. subtilis* empty vector strain (SCK6-e) was used. The scale bar represents 1 μm.

**Figure 2:**
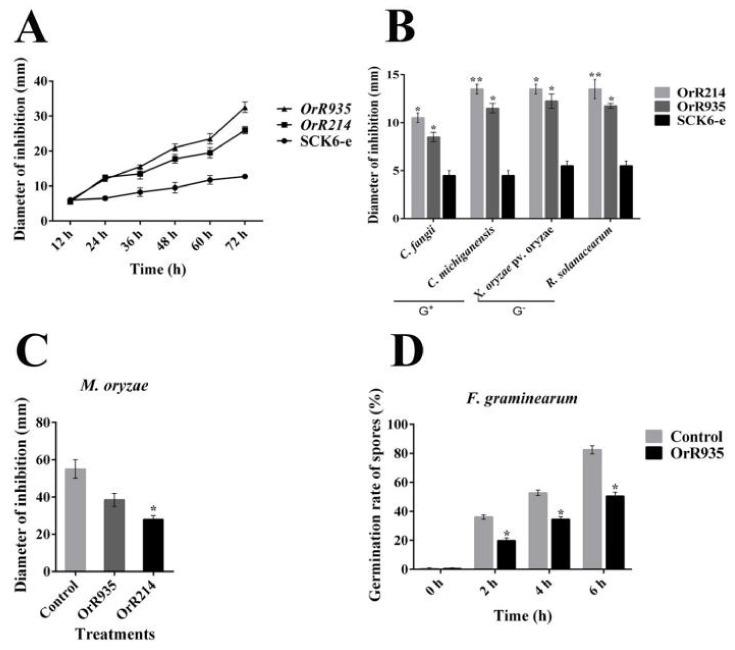
Analysis of antimicrobial potentiality of two resistance genes against pathogens. The *B. subtilis* SCK6-e strain was employed as a control in this assay. (**A**) Growth and diffusion diameter of autolytic strains. (**B**) Both groups of bacterial inhibition compared with SCK6-e in response to peptides OrR214 and OrR935. (**C**) Inhibition of OrR214 and OrR935 peptides against *M. oryzae* compared to buffer. (**D**) Inhibition of the germination rate of OrR935 peptides against spores of *F. graminearum* compared to buffer. Three individual experimental data points were collected for the mean values. SD (standard deviation) is represented by vertical bars. *T*-tests were carried out to evaluate the significance levels at * *p* ≤ 0.05 and ** *p* ≤ 0.01.

cDNA libraries were developed using the *B. subtilis* expression system to identify AMPs from *O. rufipogon* due to its highly effective expression ability. Considering the initial library titer, the libraries were tested, and the rate of recombination was 1.1 × 10^7^ CFU/mL (colony-forming units per milliliter) (Figure S1). Initially, we screened 38 monoclonal clones from a total of 3114 transformants, and phenotypic abnormalities (autolysis) were seen repeatedly. Among them, six clones exhibited strong antimicrobial properties that caused serious damage to host cells (abnormal morphology) with substantial changes in cell structure (Figure 1) due to the expression of *O. rufipogon* foreign resistance genes. Interestingly, these resistance genes not only caused *B. subtilis* cell damage but also changed their growth pattern to sparse and massive colonies compared with those without autolysis (Figure 1 and Figure 2A). These results indicate that the peptide encoded by the inserted gene is toxic to the host cell, leading to the development of autolysis with a diffusion growth pattern. After BLAST searches in the NCBI database, we found that OrR214 and OrR935 were new peptides (Table S4). For further study based on this screening, the OrR214 and OrR935 genes were chosen.

### 2.2. Antimicrobial Activity of Resistance Genes against Pathogenic and Non-Pathogenic Bacteria

The extracellular antimicrobial features were examined against Gram-positive and Gram-negative bacteria (*Clavibacter fangii*, *Xanthomonas oryzae* pv. oryzae, *Clavibacter michiganensis* subsp, and *Ralstonia solanacearum*) compared to the control SCK6-e strain (Table S3). The peptides OrR214 and OrR935 had substantial inhibition rates (Figure 2B). In addition, the biological activities against fungal pathogens were also measured (Figures S2 and S3). Among them, the OrR214 peptide could significantly inhibit the mycelium growth of *Magnaporthe oryzae* (Figure 2C), whereas the OrR935 peptide could inhibit the spore germination of *Fusarium graminearum* (Figure 2D).

### 2.3. Stabilities of Antimicrobial Peptides Influenced by Temperature and Enzymes

**Figure 3:**
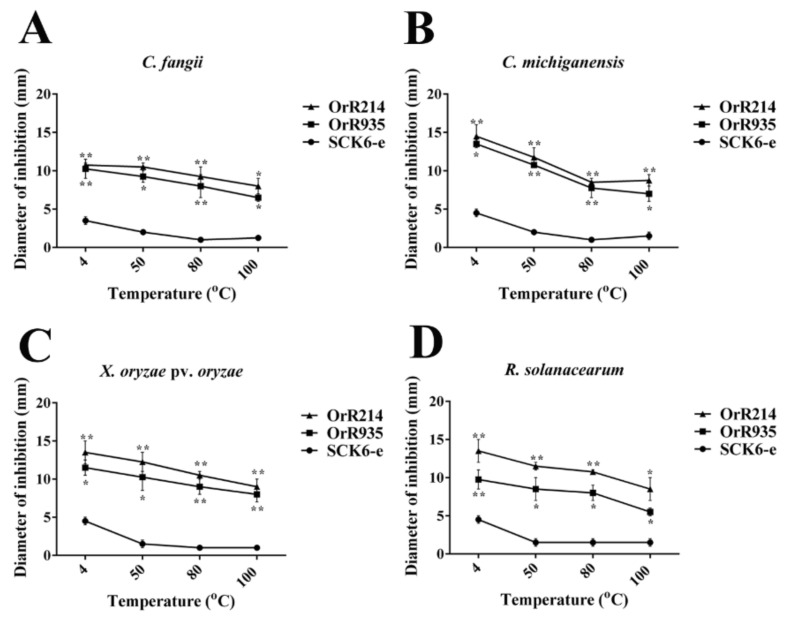
Stabilities of the antimicrobial peptides. Bacteriostatic maps of four indicator bacteria (*C. fangii*, *X. oryzae* pv. oryzae, *C. michiganensis*, and *R. solanacearum*) on the plate at different temperatures. (**A**) (*C. fangii*), (**B**) (*C. michiganensis*), (**C**) (*X. oryzae* pv. oryzae), and (**D**) (*R. solanacearum*) are graphs of the bacteriostatic diameters of two peptides at various temperatures (4 °C, 50 °C, 80 °C, and 100 °C). Three individual experimental data points were collected for the mean values. SD is represented by vertical bars. T-tests were conducted for significance analysis at * *p* ≤ 0.05 and ** *p* ≤ 0.01.

**Figure 4:**
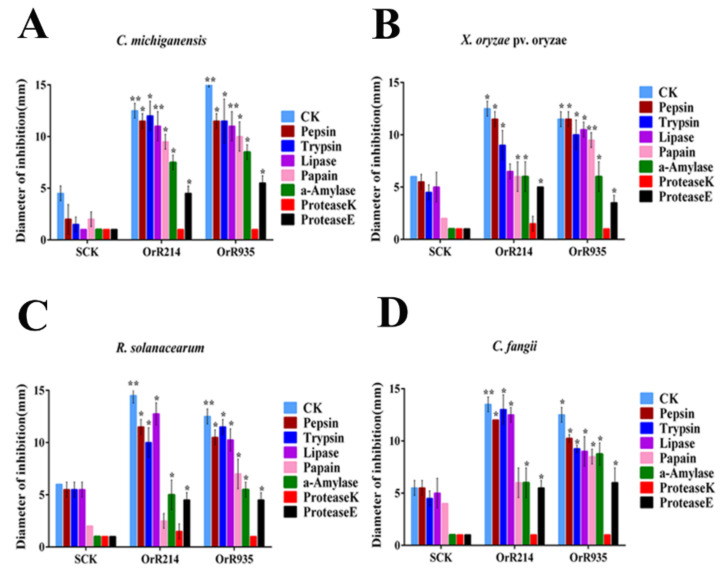
Stability tests of antimicrobial peptides. Inactivation of the antimicrobial activity of two peptides, OrR214 and OrR935, was tested by different biological enzymes. The SCK6-e *B. subtilis* strain was used as a control, and stabilities were tested against four indicator bacteria. (**A**) (*C. michiganensis*), (**B**) (*X. oryzae* pv. oryzae), (**C**) (*R. solanacearum*), and (**D**) (*C. fangii*) represent treatments with seven different kinds of biological enzymes, including pepsin, trypsin, lipase, papain, α-amylase, peptidase K, and protease E. The data represent the average of three separate experiments. *T*-tests were conducted for significance analysis at * *p* ≤ 0.05 and ** *p* ≤ 0.01.

**Figure 5:**
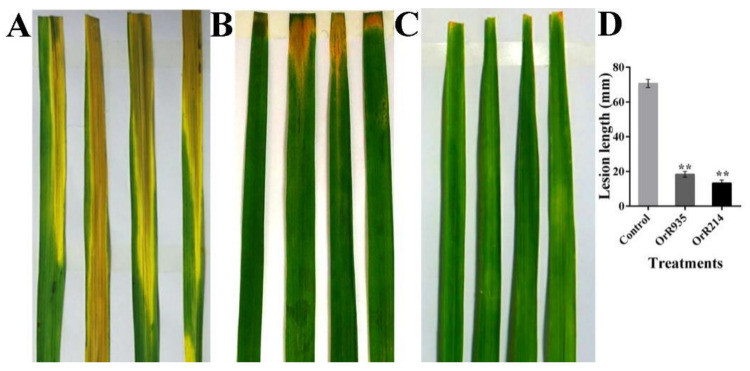
Resistance of rice leaves (*X. oryzae* pv. oryzae) treated with antimicrobial peptides. The picture shows symptoms of disease on leaves after 7 d of inoculation. (**A**) *B. subtilis* SCK6-e (Control), (**B**) OrR935, (**C**) OrR214, and (**D**) the data lesion length of two peptides OrR935 and OrR214 on rice leaves at 7 d post-inoculation. For the mean values, three individual experimental data points were collected. The vertical bars reflect SD, and the data were analyzed GraphPad Prism 6 software. T-tests were conducted for significance analysis at ** *p* ≤ 0.01.

The peptides were heated for 30 min at 4 °C, 50 °C, 80 °C and 100 °C to determine the stability of the antimicrobial activities. The results from the experiment showed that peptides OrR214 and OrR935 displayed significant activity against Gram-positive and Gram-negative bacteria at 4 to 100 °C temperature ranges compared to the control (Figure 3). After that, when tested for its sensitivity to enzymes, the results showed that the OrR214 and OrR935 peptides still have notable activity under the action of all proteases but decreased slightly after treatment with peptidase K and protease E compared to controls SCK6-e (Figure 4). The activities of the antimicrobial peptides OrR214 and OrR935 were also tested on disease-infected detached rice leaves under in vitro conditions at 7 d. Compared with the control groups (Figure 5A), two antimicrobial peptides, OrR935 and OrR214, decreased necrotic leaf lesion growth simultaneously (Figure 5B,C). The effects of two kinds of antimicrobial peptides on *X. oryzae* pv. oryzae are displayed in Figure 5D; the results showed that the incidence of rice leaves treated with antimicrobial peptides OrR214 and OrR935 decreased significantly.

### 2.4. SDS-PAGE Analysis Revealed the Peptide Expression and Sizes of OrR214 and OrR935 and Minimum Inhibitory Concentration (MIC) Assay

In *B. subtilis* SCK6, the His-tagged and TEV fusion peptides OrR214 and OrR935 were observed, and then the length of the extracellular peptides was filtered and checked by SDS-PAGE. The molecular weights of OrR214 (approximately 3.3 kDa) and OrR935 (approximately 2.3 kDa) were verified from the gel (Figure S4), and we found that the observed size was similar to the predicted size. Using the minimum dilution method, the minimum inhibitory concentration (MIC) values of OrR214 and OrR935 peptides were determined after purification against Gram-negative and Gram-positive bacteria (*C. fangii, R. solanacearum*, *C. michiganensis*, *X. oryzae* pv oryzae, *X. oryzae* pv. oryzicola, and *B. subtilis* (168)). The results revealed that the peptide OrR935 (MIC 31.4–44.0 μM) had lower antibacterial activity. In comparison, the antimicrobial potential of the OrR214 (MIC 7.7–10.7 μM) peptide against both types of bacteria was substantially higher than that of polymyxin B (MIC 5.0–25.0 μM) (Table 1).

### 2.5. Time-Kill Curve Analysis

**Figure 6:**
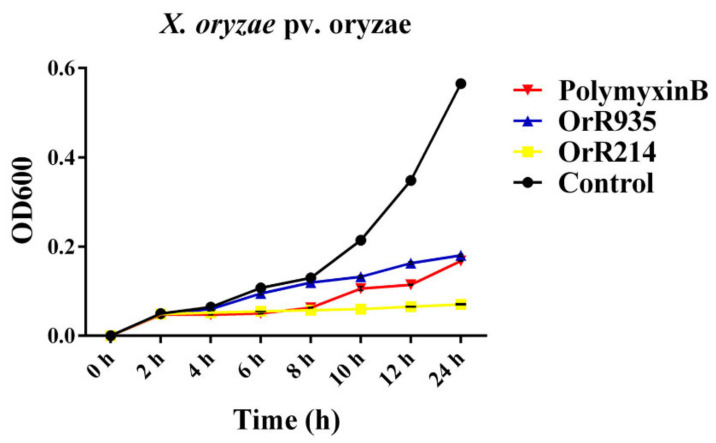
Time-Kill curve. The bacterial growth curve confirmed the inhibitory effects of the antimicrobial peptides OrR214 and OrR935. Time kill kinetics of *X. oryzae* pv. oryzae by antimicrobial peptides OrR214, OrR935, and Polymyxin B at their MICs. Three individual experimental data points were collected for the mean values.

The killing abilities of peptides OrR214 and OrR935 on bacterial cells was further studied by analyzing the growth of different Gram-positive and Gram-negative bacteria after peptide treatment at MICs. The results indicated that compared with polymyxin B (MIC 5.0 µM), with better bactericidal efficacy, *X. oryzae* pv. oryzae treated with the antibacterial peptide OrR214 (MIC 10.1 µM) hardly grew after 12 h of treatment (Figure 6). In contrast, the OrR935 peptide (MIC 44.0 µM) showed lower bacteriostatic activity against *X. oryzae* pv. oryzae. Time kill kinetics of *X. oryzae* pv. oryzicola, and *R. solanacearum* are shown in Supplementary Figure S5.

### 2.6. Assay of Hemolytic Activity of Antimicrobial Peptides Against Mammalian Cells

**Figure 7:**
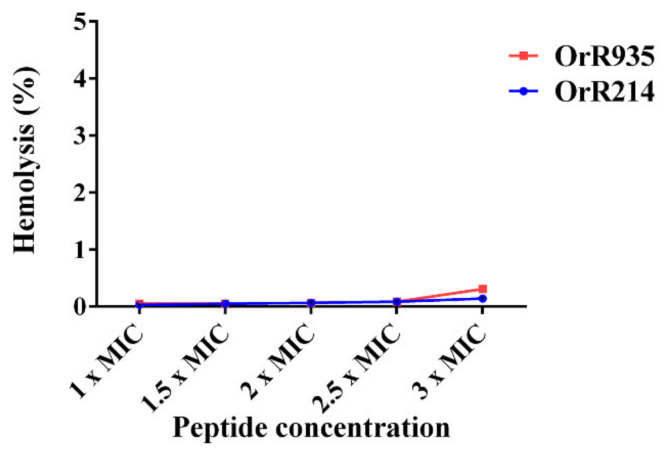
Hemolytic activity curves of the peptides on porcine erythrocytes. The release of hemoglobin was monitored at 385 nm by measuring the absorbance using a microplate reader.

The potential toxicities of two different antimicrobial peptides were then examined in a hemolytic activity assay. The hemolytic activity results revealed that peptides OrR214 and OrR935 had 0.142% and 0.306% hemolytic activity at a concentration of 3 × MICs (Figure 7). We observed similar cell survival rates (data not shown) when using higher peptide concentrations (up to 150 µM). To conclude, these two peptides were determined to be relatively safe for mammalian cells in circumstances that resemble the use of human medicines.

### 2.7. Salt Sensitivity Assay of the Antimicrobial Peptides OrR214 and OrR935

**Figure 8:**
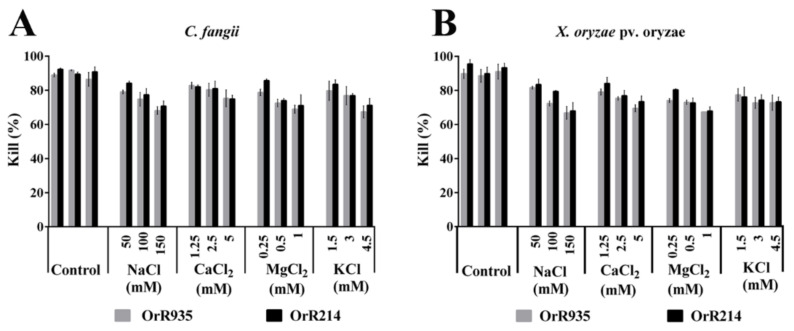
The effects of antimicrobial peptides under various cationic concentrations against the indicator bacteria *X. oryzae* pv. oryzae (**A**) and (**B**) *C. fangii*. The control contained no salt. Three individual experimental data points were collected for the mean values.

It has been reported that cations in salt solutions have a particular influence on antimicrobial peptides. For this reason, we studied the effect of cations on the peptides OrR214 and OrR935 (Figure 8A,B), and the results showed that these two AMPs had no antibacterial effect against *C. fangii* in K^+^, Ca^2+,^ and Mg^2+^ salt solutions, while the antibacterial activity against *X. oryzae* pv. oryzae was slightly inhibited. At the 50–100 mM Na^+^ concentration, these two peptides still had a significant bacteriostatic effect on the *C. fangii* and *X. oryzae* pv. oryzae. In contrast, in the presence of 150 mM Na^+^ concentration, the antimicrobial activities of these two peptides on *C. fangii* and *X. oryzae* pv. oryzae were slightly affected.

### 2.8. Antimicrobial Peptides OrR214 and OrR935 Induce Reactive Oxygen Species (ROS) Production

Antimicrobial peptides may cause a microbial reactive oxygen burst in the process of inhibiting pathogenic microorganisms, which is generally considered to be one of the mechanisms of antimicrobial peptides. The 2,7-dichlorodihydrofluorescein (DCFH) produced by 2,7-dichlorodihydrofluorescein diacetate (DCFH-DA) hydrolysis in cells can be oxidized by reactive oxygen species to produce green fluorescence. To detect the production of Reactive Oxygen Species (ROS) by the antimicrobial peptides OrR214 and OrR935, DCFH-DA was oxidized by reactive oxygen species to produce green fluorescence under a fluorescence microscope, and then the ROS levels were determined. The results are shown under the MIC conditions where two antimicrobial peptides and bacterial cells were incubated for 30 min. After that, the fluorescence intensity was significantly increased and still showed an upward trend at 120 min compared to the untreated strain (Figure S6).

### 2.9. Effects of OrR214 and OrR935 Peptides on Cell Membrane Permeability

**Figure 9:**
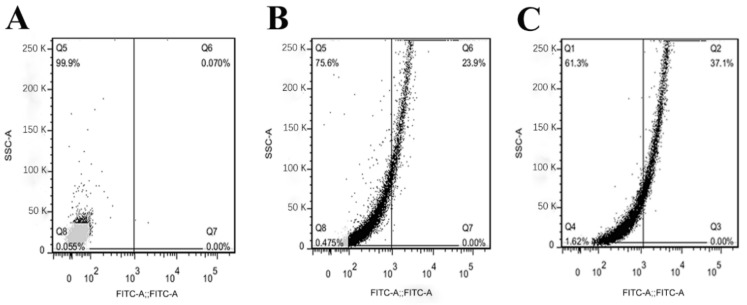
Flow cytometry analysis. Cells in the exponential phase of *X. oryzae* pv. oryzae (**A**–**C**) were treated with the peptides and analyzed for cell fluorescence by flow cytometry. The delta fluorescence signal indicates the propidium iodide (PI) uptake caused by peptide treatment. (**A**): no peptide (negative control, 0.07%); (**B**) OrR935 (MIC, 23.9%); (**C**) OrR214 (MIC, 37.1%). The *x*-axis indicates the average intensity of the fluorescence, and the *y*-axis displays the light from the side scatter. Experiments under the same conditions were replicated three times, and similar findings were noted.

To further explore the effects of OrR214 and OrR935 peptides on cell membrane integrity, propidium iodide (PI) staining was performed. PI is a nucleic acid dye that enters through a disrupted cell membrane and stains upon binding to a double-stranded nucleic acid. Dot plot analysis showed that the staining rate was significantly increased after treatment with the antimicrobial peptides OrR214 and OrR935, approximately 23.9% for OrR935 (Figure 9B) and 37.1% for OrR214 (Figure 9C), and the control was 0.07% (Figure 9A). These results suggested that peptides OrR214 and OrR935 disrupted the cell membrane integrity of the bacterial cells, and the damage to the cell membrane increased with a higher peptide concentration.

### 2.10. Antimicrobial Mechanisms of Peptides OrR214 and OrR935

**Figure 10:**
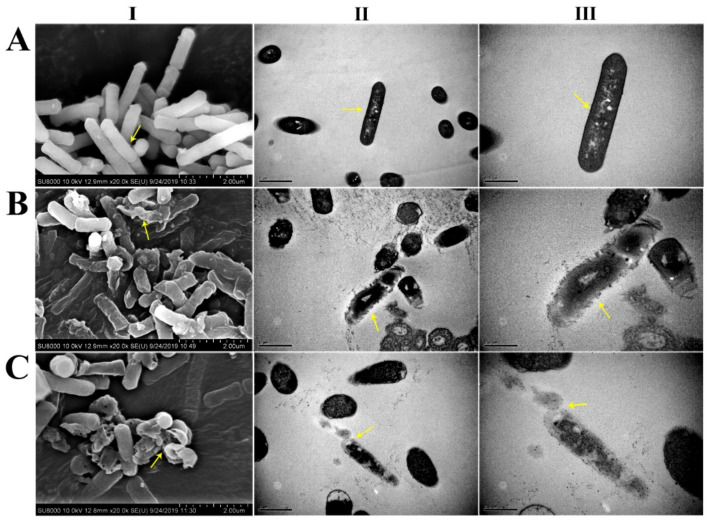
Cell membrane damage of *X. oryzae* pv. oryzae cells treated with two antimicrobial peptides (OrR214 and OrR935) at their MICs. Before the samples were coated with gold, glutaraldehyde was used for freeze-drying. (**A**-**I**) PBS, (**B**-**I**) OrR214, and (**C**-**I**) OrR935 were examined under the scanning electron microscope. (**A**-**II**) PBS, (**B**-**II**) OrR214, and (**C**-**II**) OrR935 were examined at 1 μm resolution under the transmission electron microscope. (**A**-**III**) PBS, (**B**-**III**) OrR214, and (**C**-**III**) OrR935 were examined at 0.5 μm resolution under the transmission electron microscope. Treatment with PBS was used as a control. The yellow arrows indicate major differences in different treatments.

To establish the bacteriostatic effect of antimicrobial peptides, the morphological changes in the cells were obtained by scanning electron microscopy (SEM) and transmission electron microscopy (TEM) (Figure 10). The results showed that the control had complete and smooth surfaces and no membrane damage. In comparison, the bacteria treated with antimicrobial peptide OrR214 (Figure 10BI–III) had severe damage to the cell membrane surface, and their integrity was destroyed, while the bacterial cells treated with antimicrobial peptide OrR935 (Figure 10CI–III) even had cell contents released to the cells. These findings provide a potential theoretical basis for the mechanism of cell membrane damage caused by antimicrobial peptides.

## 3. Discussion

The increasing resistance of microorganisms to traditional antibiotics is a global public health concern that needs significant attention for mitigation. Because of their broad-spectrum antibacterial and antifungal properties, the potential for AMPs as substitutes for antibiotics has increased [44]. AMPs can also function in different ways on microbial cell membranes, reducing the development of drug resistance to a certain extent [45]. *B. subtilis* has a unique ability, even at high concentrations, to secrete peptides in its surroundings, thus reducing the toxicity of host cells through its soluble secretory properties [46]. By using the *B. subtilis* expression system, we developed a new, sensitive and high-throughput technique to isolate novel AMPs [27]. Throughout this study, we identified 6 different candidate genes from a storage capacity of 3114 clones of the *O. rufipogon* cDNA library. Two distinct peptides, OrR214 and OrR935, showing significant antimicrobial properties were selected for further study. Due to the accumulation of expressed extracellular peptides, these peptides had certain toxic effects on the host cells, resulting in abnormal changes in the phenotypes of the recombinant strains (Figure 1).

At present, there are more than 4800 AMPs in the DRAMP (Data repository of antimicrobial peptides) database (http://dramp.cpu-bioinfor.org/), where the majority of peptides are effective against Gram-positive bacteria [47]. Our system provides a new strategy for screening AMPs from the above findings because it exhibited better antimicrobial activity not only against Gram-positive bacteria but also against Gram-negative bacteria. According to this study, the candidate peptides isolated encode 20–40 amino acids (Table S4). In addition, the results showed that the structures, isoelectric points (IPs) and hydrophilicities of antimicrobial peptides affect their antibacterial activity [48,49,50]. In our study, the biological information of the obtained antimicrobial peptides was also predicted. These peptides mostly contain an α-helix structure and amphiphilicity, high IPs and have some potential antimicrobial properties against Gram-positive and Gram-negative bacteria. As predicted, the peptides OrR214 and OrR935 displayed the correct molecular sizes, which can be seen from Supplementary Figure S4.

The physical interaction between the AMPs and the bacterial cell membrane has been reported to occur during the resistance process [44,51,52]. It is worth noting that during the experiment, the antimicrobial activity was partially lost due to the addition of physiological salts [53]. Some studies indicate that because AMPs contain a hydrophobic residue cation, they can interact with a negative charge on the outer membrane of the bacterial cell and thus contribute to the role of the peptide in the membrane of the bacterial cell [54,55]. In this study, when treated with a solution containing calcium, sodium and magnesium ions at low concentrations, the activity of the peptides OrR214 and OrR935 was not significantly inhibited; however, the activities of these two peptides were slightly decreased at maximum sodium ion concentrations (Figure 8A,B).

Antimicrobial genes have different effects on bacteria than traditional antibiotics. For instance, the resistance mechanism of AMPs to bacteria has also been studied. The findings are still preliminary, but it has been stated that through electrostatic interaction with the cell membrane, the cationic region of the AMPs attacks the cells, thus destroying the bacteria. Most AMPs are known to act on the bacterial plasma membrane, and there is no particular target site [56]. The confocal microscopy images and electron microscopy (both SEM and TEM) findings in our study showed damage to the cytomembrane (distortion, pore formation, shrinkage, and ruptures), leading to bacterial death, which induces PI to enter the cells and bind DNA. Meanwhile, PI can penetrate the cell membranes and release red fluorescence, which is responsible for most of the breakdown of the cell. Based on these findings, we predicted that the potential working mechanism of the OrR214 and OrR935 peptides was rupture of the cell membrane.

In addition, our findings (Figure S6) have shown that antimicrobial peptides can also induce the formation of reactive oxygen species. Most molecules in cells, including nucleic acids and proteins, may be killed by ROS, resulting in antibacterial actions, although it is difficult to establish the particular target points of action. While this finding is consistent with previous findings, further analysis is still needed to determine the unique mechanisms employed by these two peptides [57]. Even though antimicrobial peptides have broad-spectrum antimicrobial activity, their development and application still have some safety issues. Some antimicrobial peptides have been demonstrated to be toxic to mammalian cells in previous studies [58,59].

## 4. Materials and Methods

### 4.1. Maintenance of Plant and Pathogen Cultures

The China Germplasm Resources Conservation Center supplied the Zhangpu wild rice, and the seeds were immersed in a petri dish for 3 d before germination. The cultures of *Rhizoctonia solani* were taken from our laboratory. Before use, the cells were inoculated on a PDA plate and cultured in a 28 °C incubator. After 3–4 generations of activation, *R. solani* was inoculated at the tillering stage of rice, and leaves were collected every 6 h until the onset of rice [60]. All indicator bacteria used in this experiment were isolated and collected by our lab.

### 4.2. Construction of the cDNA Library and Quality Assessment

The fundamental steps of the construction of the cDNA library are as follows: total RNA extraction from leaves using the TRIzol method, mRNA purification using the PolyATtractR mRNA isolation system (Promega, Madison, WI, USA), inverting and synthesizing using a cDNA kit (Takara Biomedical Technology, Dalian, China), adding the same cleavage site to the vector at the end of cDNA by PCR and ligating with the PBE-S vector, and finally transforming to the HST08 expression system. Transformants were picked randomly from each plate for PCR detection to evaluate the quality of the cDNA library. The pBE-S-F (5′-GTTATTTCGAGTCTCTACGG-3′) and pBE-S-R (5′-TAACCAAGCCTATGCCTACA-3′) primers were used to confirm the consistency of the cDNA library colonies and were then stored at −80 °C [61]. Primers for *O. rufipogon* library construction and purified peptides are presented in Supplementary Table S2.

### 4.3. Candidate Resistance Gene Screening and Confirmation from cDNA Libraries

As with the previously mentioned method [27,43], initial screening and confirmation were carried out. The plasmid population was extracted and transferred into *B. subtilis* SCK6. All single colonies were picked and placed in 2 mL Eppendorf tubes containing kanamycin (10 mg/L) LB medium and stored at 37 °C (4–6 h, 180 rpm). After that, on the LB plate containing kanamycin, 1 μL of each single colony was placed, and the morphology of the colony was observed every 12 h. The strains with abnormal phenotypes (autolysis) were recorded and verified repeatedly.

### 4.4. Expression of the Antimicrobial Peptide Against Pathogenic Bacteria and Thermal Stability

The extracellular peptides secreted by the *B. subtilis* expression system were extracted by the ammonium sulfate precipitation method to determine the biological activity of antimicrobial peptides. For the measurement of bacterial bioresistance, an agar diffusion test (filter paper method) was used, in which the peptide was dropped on filter paper on an indicator plate [43]. Stability experiments were conducted to verify the strength of the antimicrobial peptides against different temperatures and enzymes. The antimicrobial peptides were heated for 30 min at 4 °C, 50 °C, 70 °C, and 100 °C for thermal stability testing. Then, AMPs were treated with 100 μg/mL lipase (30 °C), pepsin and trypsin (37 °C) and papain, amylase, peptidase K, and protease E (55 °C) for 1 h to measure the effects of the enzymes on the AMPs. The agar diffusion test was performed on two Gram-negative (*R. solanacearum* and *X. oryzae* pv. oryzae) and Gram-positive (*C. michiganensis* subsp and *C. fangii*) bacteria. Importantly, we confirmed that the pure antimicrobial peptides (after purification) were also stable under higher temperatures and were insensitive to enzymes.

### 4.5. His-Tag Fusion Peptide Purification

The genes OrR214 and OrR935 were engineered by a 6×His tag purification process, and a segment of the protease (TEV) sequence was inserted between the vector (PBE-S) signal peptide and the target fragment and then transformed into a B. subtilis expression system. The detailed methods and recombination peptide sequences for obtaining pure AMPs are presented in Supplementary Method 1 and Table S1. TEV protease is a highly specific cysteine protease that recognizes the amino-acid sequence Glu-Asn-Leu-Tyr-Phe-Gln-(Gly/Ser) and cleaves between the Gln and Gly/Ser residues. This protease is commonly used to remove His-Tags of fusion proteins. There was only one additional Gly/Ser amino acid residue at the N end of the target protein after His-label was digested, thus decreasing the effects on the structure and function of the target protein and obtaining pure peptides. The remaining purified peptide was digested with TEV protease at 4 °C overnight. To remove TEV (His-Tag), the solution was passed through a Ni-NTA His Bind Resin. The peptide was concentrated in an ultrafiltration tube, and then the solvent was exchanged with PBS. Finally, the peptides were concentrated and recovered for subsequent experiments. First, 40 μL of purified peptide was boiled for 10 min in a water bath with 10 μL of 5× peptide loading buffer. Next, a Tricine-SDS-PAGE kit was used to resolve 25 μL of the mixed sample onto a polyacrylamide gel using a Tricine-SDS-PAGE kit. A list of specific primers for obtaining pure AMPs and detailed purification method was presented in Supplementary Table S1 and Method S1.

### 4.6. Minimum Inhibitory Concentration (MIC) and Growth Time-Kill Curve Analyses

A microtiter broth dilution method [62,63] was used to determine the MICs of antimicrobial peptides. For these tests, the bacterial inoculum was prepared in LB liquid medium (OD600 ≈ 0.02–0.05) and added to a 96-well titration plate. Serial dilutions of equal amounts of peptides were then performed, and the plates were incubated at 28 °C for 24 h. The lowest peptide concentration of bacteria was recorded without visible growth at the MIC value using ELISA (SPARK). The concentration of purified antimicrobial peptides was modified to the MIC level for determination of time-kill analysis, and the same volume as the indicator bacteria was applied to the 96-well plate (at least three wells per concentration). Then, the plate was mixed and incubated for 2, 4, 6, 8, 10, 12, and 24 h at 28 °C. After that, the OD was measured at 600 nm. Inhibition assays for *M. oryzae* were performed as described previously with slight modifications [64]. Nearly 200 μL of peptide was mixed with 5 mL of semisolid PDA media for a final concentration of 260 μg/mL and was spread on previously prepared PDA plates. The plates were then seeded with 5 × 5 mm mycelial plugs collected from the edges of 4 d *M. oryzae* colonies (P131), followed by incubation at 28 °C. The buffer was used as a control, and the data were recorded at 96 h. First, approximately 200 μL of peptides was added into PDA media with hyphae of *F. graminearum* and then cultured at 28 °C for 72 h. Second, the antimicrobial peptides were mixed evenly with the spores of *F. graminearum* (10^7^/mL) and cultured at 28 °C. The number of spore eruptions was observed and recorded every 2 h.

### 4.7. Salt Dependence Test

The steps of these tests are similar to the previous MIC determination except for the treatment method of the antimicrobial peptides. To detect the activity of the antimicrobial peptide, the solution (1 × MIC) was treated with different concentrations of NaCl (50, 100, and 150 mM), CaCl_2_ (1.25, 2.5, and 5 mM), MgCl_2_ (0.5, 1, and 2 mM), or KCl (1.5, 3, and 4.5 mM), and the OD values were detected at a wavelength of 600 nm using ELISA (SPARK) [65,66].

### 4.8. Hemolytic Assay

The hemolytic activity of the two antimicrobial peptides was evaluated according to a previously described method [58]. Briefly, freshly prepared porcine erythrocyte cells were centrifuged (1500× *g*) for 10 min at 4 °C and the cells were harvested, washed three times with precooled PBS solution and diluted with PBS. An equal volume of diluted red blood cells and antimicrobial peptide solutions (1 × MIC, 1.5 × MIC, 2 × MIC, 2.5 × MIC, and 3 × MIC) were incubated for 1 h at 37 °C and centrifuged at 4000× *g* for 10 min. Using an ELISA (SPARK) plate reader, the supernatant was taken in a 96-well plate, and the absorbance was measured at 385 nm. A 0.1% Triton X-100 erythrocyte suspension was used as a control group, while a mixture incubated only with PBS buffer was used as a negative control. In addition, the percentage of hemolysis was determined with 0.1% Triton X-100 in PBS. The percentage of hemolytic activity was calculated as follows: [(A_peptide_−A_PBS_)/(A_Triton_−A_PBS_)] × 100.

### 4.9. Reactive Oxygen Species (ROS) Determination Test

The fluorescence assay of DCFH-DA was used to measure the production of ROS [67]. In this experiment, *X. oryzae* pv. oryzae was cultured in liquid LB medium in the logarithmic phase, and the same volumes of OrR214 and OrR935 (MIC) antimicrobial peptides were applied. Then, 10 μM of DCFH-DA was added to the medium and incubated at 28 °C for different periods (15, 30, 60, 90, and 120 min). Samples were centrifuged at 5000× *g* for 10 min following incubation. The pellet was rinsed 2–3 times with PBS, and the cell pellet was resuspended in PBS. An ELISA (SPARK) microplate reader (excitation 485 nm and emission 540 nm) measured the fluorescence intensity of the resuspended cells and observed them with a fluorescence microscope to capture images.

### 4.10. Detection of Cell Membrane Permeability

The membrane permeability of the cells treated with the antimicrobial peptides was examined by confocal microscopy and flow cytometry [27,68]. The bacterial cells were cultured to the logarithmic growth phase, and the concentration was adjusted to OD_600_ (0.1–0.2) and incubated with antimicrobial peptides OrR214 and OrR935 at the MICs for 2 h at 28 °C. After centrifugation, the pellet was washed 2–3 times with PBS, resuspended, treated with 5 μL of PI, and the sample was then incubated at 4 °C for approximately 15 min in the dark. Then, the pellet was centrifuged, washed 2–3 times with PBS, and resuspended in 200 μL of PBS for FOW cell detection. Fluorescence intensity images were acquired using a FACSVerse machine (BD, USA) and analyzed using FlowJo. 7.6.1, Min software (BD, USA).

### 4.11. Electron Microscopy Analysis

The cells were pelleted by shaking the *X. oryzae* pv. oryzae at 28 °C to the logarithmic growth phase. The antimicrobial peptides with MICs were co-incubated for 2 h at 28 °C, and the autolyzed strains (OrR214 and OrR935) were shaken at 37 °C for 60 h and then centrifuged at 1000× *g* for 5 min. After washing 2–3 times with sterilized PBS (pH 7.4), the cells were resuspended and obtained an OD of 0.8 (at 600 nm). The cells were then extracted at 5000× *g* for 5 min by centrifugation, resuspended in 2.5% glutaraldehyde to stabilize the cell pellet for 2–4 h and rinsed thoroughly with PBS twice and with different gradients of ethanol (30%, 50%, 70%, and 90%). Finally, the samples were washed with 100% ethanol for approximately 10 min, dried overnight in a freeze dryer and inspected on a HITACHI S-4800 SEM [69]. For transmission electron microscopy analysis, the obtained cells were centrifuged (each treatment with 3% glutaraldehyde for 2 h) and then washed 3 times with 0.1 M phosphate buffer (pH 7). The cells were further fixed with 1% osmium tetroxide, and then samples were washed 3 times with sterile distilled water and stained with 2% uranyl acetate solution. The stained samples were eluted with different concentrations of ethanol (70%, 80%, 90%, and 100%), with each step requiring 15 min. The dehydrated sample was soaked with 100% propylene oxide for 15 min, washed two times, and the epoxy resin and propylene oxide were embedded at 1:1 for 1 h. The embedded sample was placed in a mold, freeze-dried at −80 °C for 12 h, sliced by an ultrathin microtome, and observed under a HITACHI H-7650 transmission electron microscope.

### 4.12. Pathogenicity Assay

To assess the pathogenicity of *X. oryzae* pv. oryzae, the method described in the previous article was used with minor modifications [70]. Leaves 10 cm long were cut with sterile scissors. The cells were pelleted by shaking the *X. oryzae* pv. oryzae at 28 °C to logarithmic growth phase, and the same volume of antimicrobial peptides was added. Then, the mixture was dipped with sterile scissors, and the tip of the leaf was cut off and cultured at 28 °C for 7 d. Each treatment was performed on 4 leaves and repeated 3 times.

## 5. Conclusions

Two novel antimicrobial peptides with broad-spectrum antibacterial activities from *O. rufipogon* were screened in this study. These two peptides have high thermal, enzymatic, and cationic stabilities and are nontoxic to mammalian cells. In addition, we also found that these two antimicrobial peptides can act on the cell membrane, causing damage. Based on these distinguishing features, we speculated that these two antimicrobial peptides could be used as promising raw materials for drug development.

## Figures and Tables

**Table 1:** MIC values (µM) of OrR214 and OrR935 peptides against various Gram-positive and Gram-negative bacteria.

Strain	MIC (µM)		
	Polymyxin B	OrR214	OrR935
*C. fangi*	25.0	10.7	37.7
*C. michiganensis*	12.5	10.5	34.6
*X. oryzae* pv. *oryzae*	5.0	10.1	44.0
*R. solanacearum*	7.5	8.1	33
*X. oryzae* pv. oryzicola	10.0	7.7	31.4
*B. subtilis* (168)	15.0	8.3	36.1

Note. Gram-positive bacteria: *C. fangii*, *C. michiganensis,* *B. subtilis* (168). Gram-negative bacteria: *R. solanacearum*, *X. oryzae* pv. oryzae, *X. oryzae* pv. oryzicola. Polymyxin B was used as a positive control.

## Supplementary Materials

The supplementary material for this article can be found online https://www.mdpi.com/1422-0067/21/22/8722/s1, Figure S1. Insertion of the cDNA library of *B. subtilis* system, Figure S2. The germination rate of *F. graminearum* spore, Figure S3. The antimicrobial peptide inhibitory activity on the *M. oryzae* spore, Figure S4. Detection of fusion peptides via SDS-PAGE, Figure S5. Analysis of Time-Kill curve, Figure S6. Antimicrobial peptides OrR214 and OrR935 caused the reactive oxygen species production in *X. oryzae* pv. oryzae cells, Table S1. Sequences for His-Tag Fusion Peptide, Table S2. Primers and sequences for building the *O. rufipogon* library and purifying the proteins, Table S3. The bacteriostatic spectrum of the antimicrobial genes of *O. rufipogon*, Table S4. Bioinformatics prediction of antibacterial peptide, Method S1: His-tag fusion peptide purification.
