# Supplementary material for: Isolation of Antimicrobial Genes from Oryza rufipogon Griff by Using a Bacillus subtilis Expression System with Potential Antimicrobial Activities
Source: Int J Mol Sci. 2020 Nov 18;21(22):8722. doi: 10.3390/ijms21228722 (PMC7698926; doi:10.3390/ijms21228722)
Supplement: Supplementary file 1 [file ijms-21-08722-s001.pdf]

Supplementary Materials

Isolation of antimicrobial genes from *Oryza rufipogon* Griff by using a *Bacillus subtilis* expression system with potential antimicrobial activities

Jiale Li, Md. Samiul Islam, Pengfei Guo, Xiaoqing Hu, Wubei Dong*

Method S1 His-Tag Fusion Peptide Purification

First, we constructed the recombinant plasmid through the following steps: design primers according to the inserted sequence of the target gene to remove PolyA tail; design the complementary oligos to the end of the insert (The downstream primer R' containing the *Xba* I restriction site and the stop codon. The upstream primer is the original vector primer pBE-S-F); the double digestion was made by two restriction enzymes named *Nde* I and *Xba* I*;* after washing put it into the PCR system under the following conditions: 95 °C for pre-denaturation for 5 min; 95 °C, 30 sec; 57 °C, 30 sec; 72 ℃, 1 min; 28 cycles; and finally extension at 72 ℃ for 10 min. After checking the PCR product by running a gel for ensuring the digestion was successful, transform the recombinant plasmid into *E. coli* DH5α. Finally, extract the plasmids from overnight cultures. Second, the synthetic target fragment containing the *Eco*52I-HIS-TEV-*Nde*I was used for constructing the recombinant strains. The double digested recombinant plasmids and the target fragments were mixed at a ratio of 3:1-10:1 at 16 °C for ligation. Then transfer it into the *E. coli* DH5α. After overnight incubation, pick transformants for PCR detection, and extract plasmids. Then, transform the plasmid into *Bacillus subtilis* SCK6 and Pick transformants and add 50% glycerol for preservation. For Ni-NTA purification, place the modified resistant strain and empty vector strain in the LB plate for overnight culturing at 37 °C. After incubation, pick the

colonies and inoculate them into 300 mL LB (containing 10 mg/L Kanamycin) medium and shake the culture at 37 ° C for 72 h. Take 600 mL fermentation broth, centrifuge at 10,000 r/min, 4 ℃ for 25 min, discard the bacteria and collect the supernatant. Thereafter, put the gasket on the bottom of the column, take Ni-NTA His-Bind-Resin and mix the packing with 20% ethanol with a pipette tip and use the pipette tip to draw 1.5 mL of the suspension into the column. Balance the column, and press the gasket above the column. Then, wash the column with a large amount of sterilized water (10 times - 20 times the column volume). After rinsing, at least 10 times equilibrate the column with a binding buffer (natural flow rate). Pass the supernatant filtrate slowly through the column with a hanging bottle (generally 4-5 times). After sample loading, wash away residual samples for 10 times. Use imidazole with different concentration gradients. Wash the column with distilled water (10 times - 20 times the column volume). Seal both ends of the purification column. If it is not used for a long time, it can be sealed by adding about 4 mL of 20% (sterilized) ethanol and stored at 4 °C. To remove TEV (His-Tag), the solution was passed by the Ni-NTA His-Bind-Resin, the remaining purified peptide was digested with TEV protease at 4 ℃ overnight. Finally, the peptides were concentrated and recovered for subsequent experiments.

Table S1 Sequences for His-Tag Fusion Peptide

Recombination antimicrobial peptide	Gene sequence
OrR214	GGCCGGTGCACATCACCATCATCACCACGAAAAC CTGTATTTTCAGTCCCATATGCTAGTAGATATATA TACACATGTGTACAATTGTACAAGTAGTGAGAAA CACACGCATTGCTATGAAATACGAAAGTCAATTTC C TCTAGATAACATCACCATCATCACCAC

OrR935	GGCCGGTGCACATCACCATCATCACCACGAAAAC CTGTATTTTCAGTCCCACATATGCTAGGCGTGCCA GTGAGCTCGACTTTGCGGTTAAATAACACGACAAT GAATCCCTGTTTGCCATCCTCTAGATAACATCACC ATCATCACCAC

Table S2 Primers and sequences for building the *O. rufipogon* library and purifying the proteins

Primer	Sequence
Oligo dT (contains *Xba* Ⅰ enzyme site)	ACAGGCTCTAGAGCTTTTTTTTTTTTTTTTTTTTTT
PBE-S-F	GTTATTTCGAGTCTCTACGG
PBE-S-R	TAACCAAGCCTATGCCTACA
214R	GCTTCTAGATTAGGAAATTGACTTTCGT ATTTCATAGCAATGCG
935R	ATATCTAGATTAGGATGGCAAACAGGGATTCAT TGTCG
F1	GGCCGGTGCACATCACCATCATCACCACGAA AACCTGTATTTTCAGTCCCA
R1	TATGGGACTGAAAATACAGGTTTTCGTGGTG ATGATGGTGATGTGCACC

Note: 214R and 935R are downstream sequences where the Poly A and His-tag of the original strain were cut. F1 and R1 are the sequences where the target segment (*Nde* Ⅰ-His-tag-TEV-*Xba* Ⅰ) was inserted.

Table S3 The bacteriostatic spectrum of the antimicrobial genes of *O. rufipogon*

Gene	***G***+		***G***-	
	C. fangii	*C. michiganensis*	*X. oryzae* pv. oryzae	*R. solanacearum*
SCK6-e	0.53 ±0.033	0.50 ±0.057	0.66 ±0.033	0.75 ±0.050
OrR214	1.1 ±0.033 **	1.2 ±0.033**	1.3 ±0.058**	1.3 ±0.050*
OrR320	0.93 ±0.088*	-	1.1 ±0.088*	-
OrR361	-	-	-	1.0 ±0.050*
OrR1135	1.1 ±0.060**	1.2 ±0.033**	1.2 ±0.11*	1.4 ±0.050*
OrR1006	1.0 ±0.050*	-	-	-
OrR169	-	-	-	-
OrR894	0.96 ±0.06*	1.0 ±0.088**	1.0 ±0.028**	-
OrR317	1.1 ±0.12*	0.90 ±0.057**	1.4 ±0.12**	1.2 ±0.075*
OrR573	1.0 ±0.050*	-	1.0 ±0.050*	-
OrR119	1.1 ±0.10**	1.1 ±0.057**	1.2 ±0.13*	1.4 ±0.10*
OrR583	-	1.2 ±0.0577**	-	-
OrR160	1.1 ±0.10*	-	0.65 ±0.050*	-

OrR935	1.0 ±0.057*	1.0 ±0.058**	1.20±0.057**	1.25±0.050*
OrR561	-	-	-	-
OrR313	1.1 ±0.066*	1.1 ±0.057**	1.0±0.088*	1.4 ±0.10*
OrR874	1.2 ±0.10*	-	1.2±0.050*	-
OrR194	-	-	-	-

Note, "-" no significant difference; * indicates significant difference ($p \leq 0.05$), ** indicates an extremely significant difference ($p \leq 0.01$). The data in the table are mean (cm) ± standard deviation (cm) of bacteriostatic diameter mean. The result is from three independent experiments. Significance analysis was performed using a t-test.

Table S4 Bioinformatics prediction of candidate antimicrobial peptides

antimicrobial peptide	AA	Amino acid sequence	Second structure	PI	MV
OrR214	28	LVDIYTHVYNCTSSEKHTHCYEIRKSIS	α-helix Random coil	7	3327.0
OrR119	80	RKVARERERSVSLLSGSRSGRCWRSCGTTWWPVLDRRLASRSSARPLQPAPLSSTKMATARRAEWRTSGRSRCRRPRRRR	α-helix Random coil	12.24	9351.7
OrR317	46	LLVWTVQIDRRSMKHLSVSQCHVQCTFKFIWN KIGPWTVYHWAVRQ	α-helix Random coil	10.06	5627.6

OrR313	32	LVNYRRVGRIKPVHSDLLIGRTKCLD LWLR	α-helix Random coil	10.89	3591.3
OrR935	22	LGVPVSSTLRLNNTTMNPCLPS	Random coil	8.25	2314.7
OrR1135	25	LSFCSCIRTNLDGFDQKLQWNPHFY	α-helix Random coil	6.73	3033.4

Note, AA No.: number of amino acids, PI: theoretical pI, MV: molecular weight.

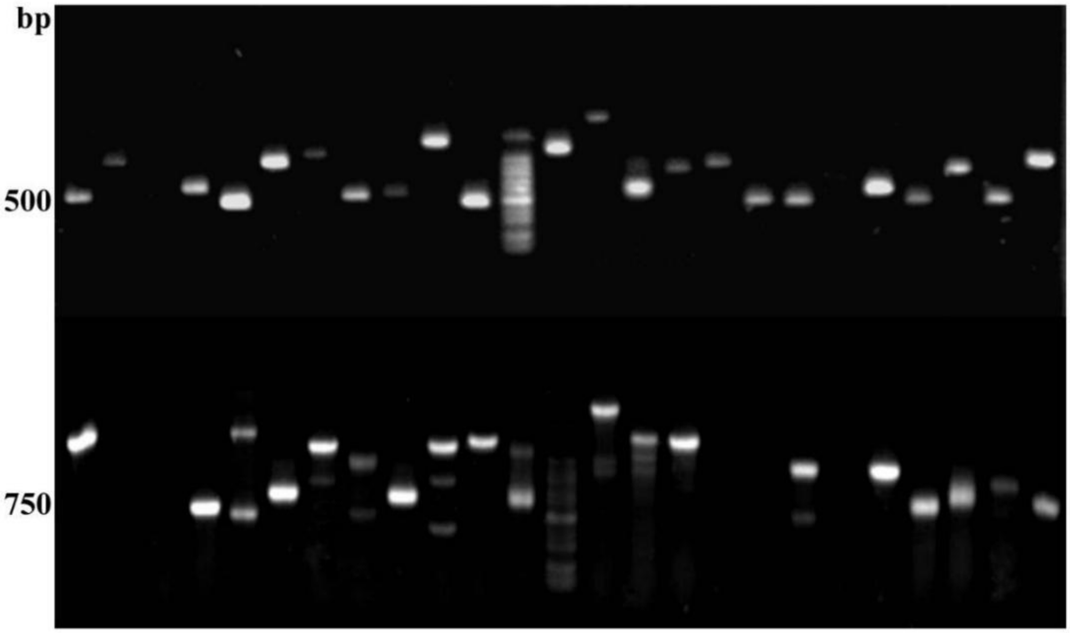

Figure S1. Insertion of the cDNA library of *B. subtilis* system. The titer of the library was 1.1 $\times 10^7$ pfu/ml, and the marker was 100 bp.

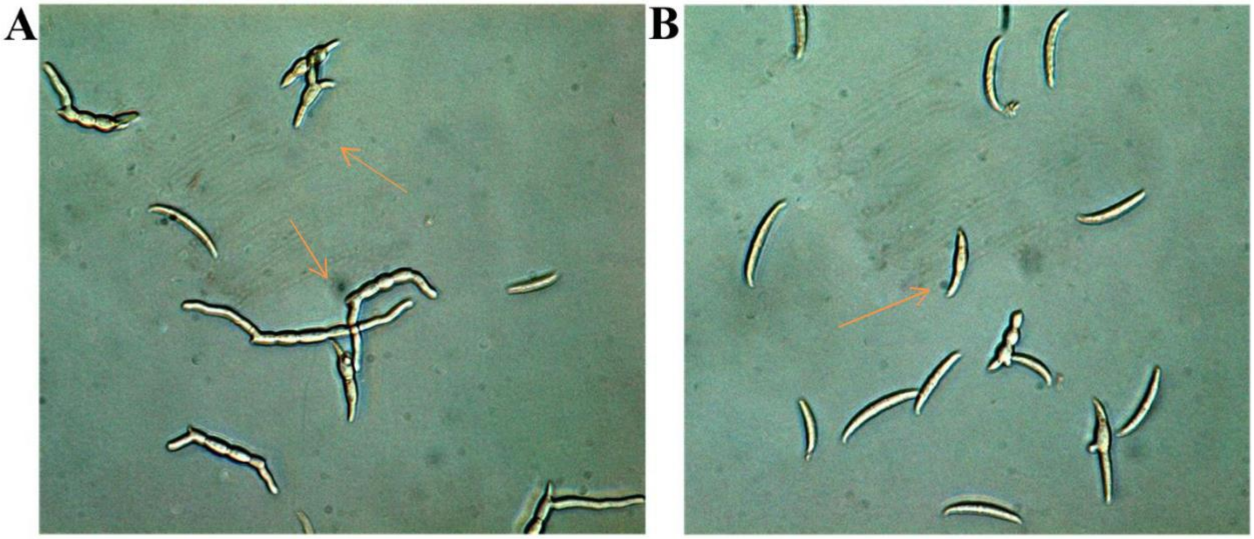

Figure S2. The germination rate of *F. graminearum* spore was significantly inhibited by the antimicrobial peptide OrR935. The antimicrobial peptide OrR935 and the PBS buffer (control) were co-incubated with spores for 4 h and observed under the light microscope (40 ×). (**A)** PBS buffer and **(B)** antimicrobial peptide OrR935.

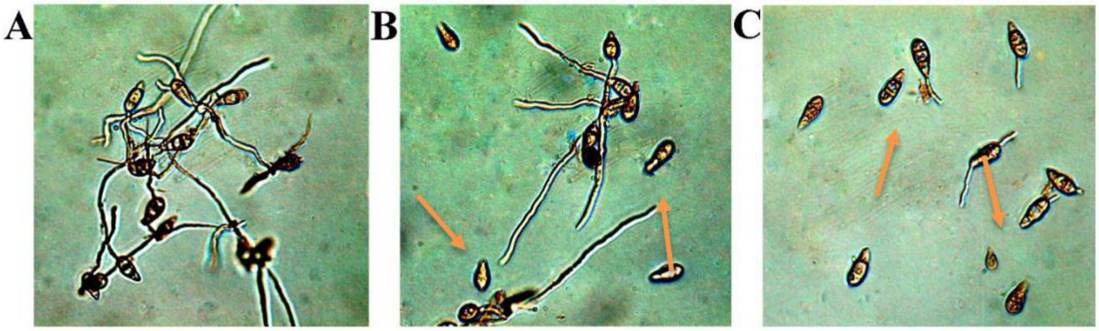

Figure S3. The antimicrobial peptide inhibitory activity on the *M. oryzae* spore. The antimicrobial peptide was co-incubated with spores for 6 h and observed under the electron microscope (40 ×). (**A)** PBS buffer, (**B)** antimicrobial peptide OrR935, and (**C)** antimicrobial peptide OrR214, where PBS buffer as control.

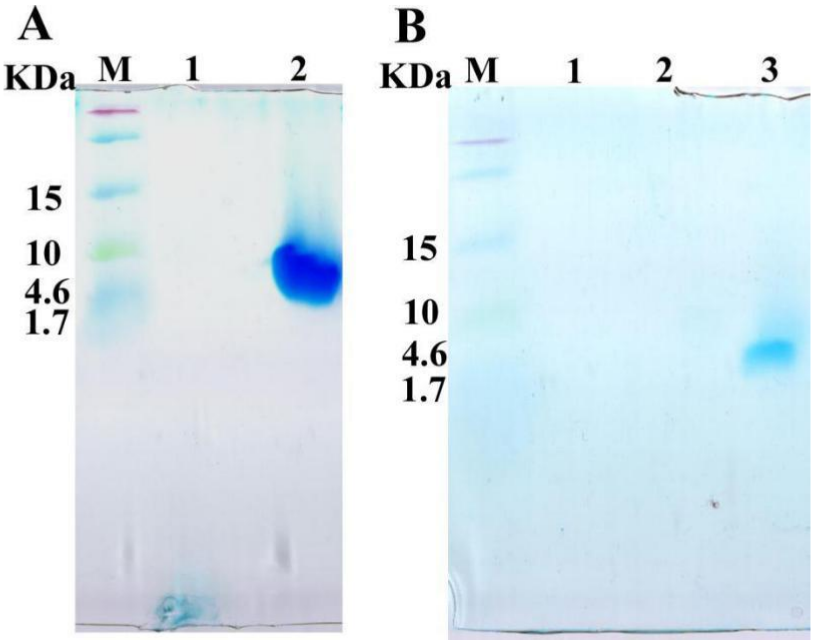

Figure S4. Detection of fusion peptides via SDS-PAGE. Lane M indicates the pre-staining ultra-low molecular weight (1.7-40 kDa) marker. Lane **A-1** and **B-1** are the control of PBS buffer. Lanes **A-2**, **B-2**, and **B-3** indicate the molecular weight of the peptide OrR214 (approximately 3.4 kDa), SCK6-e (empty vector), and the peptide OrR935 (approximately 2.3 kDa), respectively.

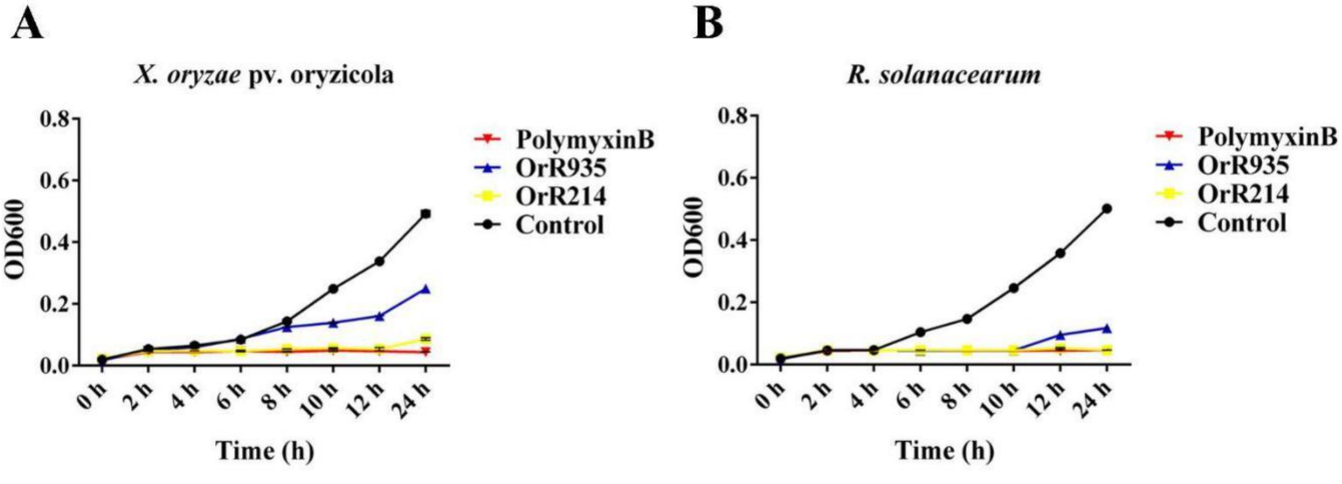

Figure S5. Analysis of Time-Kill curve. The inhibitory effects of the antimicrobial peptides OrR214 and OrR935 were confirmed by bacterial growth curves. Killing kinetics of *X oryzae* pv. oryzicola (**A)** and *R. solanacearum* (**B)** by antimicrobial peptides OrR214, OrR935 and Polymyxin B at their MIC concentrations; the Polymyxin B was used as a positive control.

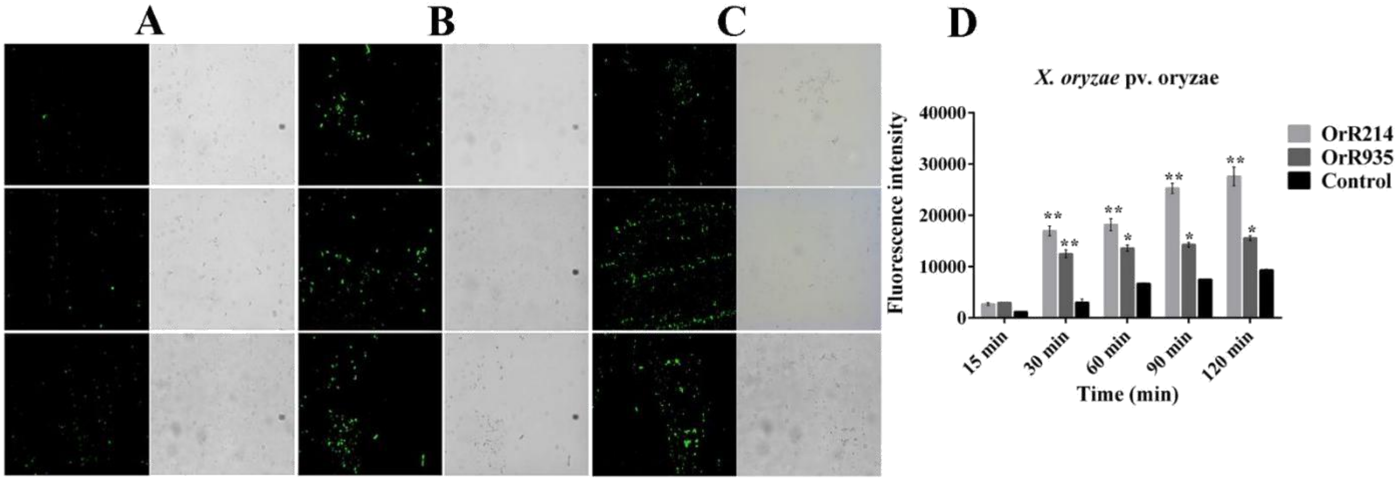

Figure S6. Antimicrobial peptides OrR214 and OrR935 caused the reactive oxygen species production in *X. oryzae* pv. oryzae cells. From (**A**) to (**C**), pictures represent fluorescence intensity, (**A**) PBS buffer (control), (**B**) Antimicrobial peptide OrR214, and (**C**) Antimicrobial peptide OrR935. (**D**) was the burst of reactive oxygen species at different time points. Data were the average of three independent experiments, "*" $p \le 0.05$, "**" $p \le 0.01$.
